# Is Decreased Xylem Sap Surface Tension Associated With Embolism and Loss of Xylem Hydraulic Conductivity in Pathogen-Infected Norway Spruce Saplings?

**DOI:** 10.3389/fpls.2020.01090

**Published:** 2020-07-16

**Authors:** Teemu Paljakka, Kaisa Rissanen, Anni Vanhatalo, Yann Salmon, Tuula Jyske, Nønne L. Prisle, Riikka Linnakoski, Jack J. Lin, Tapio Laakso, Risto Kasanen, Jaana Bäck, Teemu Hölttä

**Affiliations:** ^1^ Faculty of Agriculture and Forestry, Institute for Atmospheric and Earth System Research/Forest Sciences, University of Helsinki, Helsinki, Finland; ^2^ Faculty of Science, Institute for Atmospheric and Earth System Research/Physics, University of Helsinki, Helsinki, Finland; ^3^ Natural Resources Institute Finland (Luke), Espoo, Finland; ^4^ Nano and Molecular Systems Research Unit, University of Oulu, Oulu, Finland; ^5^ Natural Resources Institute Finland (Luke), Helsinki, Finland; ^6^ Forest Sciences/Faculty of Agriculture and Forestry, University of Helsinki, Helsinki, Finland

**Keywords:** embolism, *Endoconidiophora polonica*, hydraulic conductivity, *Picea abies* (Norway spruce), plant-pathogen interactions, surface tension, xylem transport, xylem sap composition

## Abstract

Increased abiotic stress along with increasing temperatures, dry periods and forest disturbances may favor biotic stressors such as simultaneous invasion of bark beetle and ophiostomatoid fungi. It is not fully understood how tree desiccation is associated with colonization of sapwood by fungi. A decrease in xylem sap surface tension (σ_xylem_) as a result of infection has been hypothesized to cause xylem embolism by lowering the threshold for air-seeding at the pits between conduits and disruptions in tree water transport. However, this hypothesis has not yet been tested. We investigated tree water relations by measuring the stem xylem hydraulic conductivity (K_stem_), σ_xylem_, stem relative water content (RWC_stem_), and water potential (Ψ_stem_), and canopy conductance (g_canopy_), as well as the compound composition in xylem sap in Norway spruce (*Picea abies*) saplings. We conducted our measurements at the later stage of *Endoconidiophora polonica* infection when visible symptoms had occurred in xylem. Saplings of two clones (44 trees altogether) were allocated to treatments of inoculated, wounded control and intact control trees in a greenhouse. The saplings were destructively sampled every second week during summer 2016. σ_xylem_, K_stem_ and RWC_stem_ decreased following the inoculation, which may indicate that decreased σ_xylem_ resulted in increased embolism. g_canopy_ did not differ between treatments indicating that stomata responded to Ψ_stem_ rather than to embolism formation. Concentrations of quinic acid, myo-inositol, sucrose and alkylphenol increased in the xylem sap of inoculated trees. Myo-inositol concentrations also correlated negatively with σ_xylem_ and K_stem_. Our study is a preliminary investigation of the role of σ_xylem_ in *E. polonica* infected trees based on previous hypotheses. The results suggest that *E. polonica* infection can lead to a simultaneous decrease in xylem sap surface tension and a decline in tree hydraulic conductivity, thus hampering tree water transport.

## Introduction

Trees respond to abiotic stress factors (e.g. heat, water deficit) by controlling water use and photosynthetic production with their leaf stomata, in addition to modifying growth rate and use of stored carbohydrates. Pre-existing water stress may also predispose trees to pests and pathogens (Netherer et al., 2015; Hossain et al., 2018; Matthews et al., 2018). Bark beetles are major pests of conifers in Europe (Kautz et al., 2017), and they simultaneously vector several species of ophiostomatoid fungi (Perry, 1991; Linnakoski et al., 2012; Linnakoski et al., 2016). Some of these ophiostomatoid species are likely interfering with tree water transport in the xylem. However, the mechanism by which these pathogen infections disturb tree water transport and their role in weakening tree vigor during an invasion remain unclear (Kuroda, 2005; Linnakoski et al., 2012; Giordano et al., 2013).

Xylem conduits can become air-filled, i.e. embolized, typically by air-seeding (Tyree and Sperry, 1989). Embolism reduces the ability to transport water in xylem, i.e. lowers the tree hydraulic conductance. In extreme cases, embolism may even lead to a feedforward cycle of loss in hydraulic conductance, resulting in hydraulic failure and subsequent tree mortality (McDowell et al., 2008; Brodribb and Cochard, 2009). Air-seeding occurs when the pressure difference over a water-air interface in the bordered pit membrane becomes larger than the xylem sap surface tension (σ_xylem_) force required to restrict the air bubble (Sperry and Tyree, 1988; Delzon et al., 2010; Jansen et al., 2012). Thus, with lower σ_xylem_ a conduit is embolized already at higher Ψ_stem_. The threshold for air-seeding is given by the Young-Laplace equation (Tyree and Zimmermann, 2002)

(1) ΔP=2σcosα/r,

where ΔP is the difference in pressure between a water-filled and an air-filled conduit, σ is surface tension, α is the contact angle, and *r* is the radius of the pores that separate the air-water meniscus in the pit membranes between adjacent conduits (Sperry and Tyree, 1988). The theoretical framework thus suggests the first chain in the events should be the decrease in σ_xylem_, followed by embolism formation and a decrease in hydraulic conductivity.

**Figure 1:**
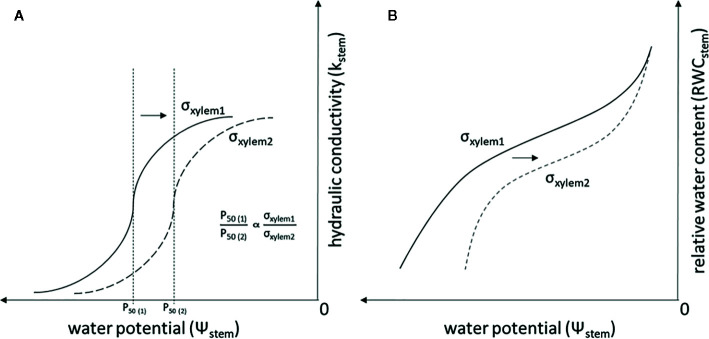
Theoretical relationship of σ_xylem_, K_stem_ and Ψ_stem_ **(A)**, and the theoretical relationship of σ_xylem_, RWC_stem_ and Ψ_stem_ **(B)** when σ_xylem_ is changed. Symbols refer to surface tensions of pure water (σ_xylem1_) and xylem sap with lowered surface tension (σ_xylem2_), P_50 (1)_ is the P_50_ value (i.e. the Ψ_stem_ where 50% of the conductivity in water transport is lost because of embolism) when the xylem sap surface tension corresponds to σ_xylem1_, and P_50 (2)_ is the P_50_ value when the xylem sap surface tension corresponds to σ_xylem2_. The relationship between P_50_ and σ_xylem_ is linear and is demonstrated also for conifers in Cochard et al. (2009).

σ_xylem_ has typically been thought to equal the surface tension of pure water. However, the variability of σ_xylem_ in natural conditions has recently received attention (Christensen-Dalsgaard et al., 2011; Domec, 2011; Schenk et al., 2015; Losso et al., 2017; Schenk et al., 2017). A decrease in σ_xylem_, e.g. due to surfactants, has been shown to increase xylem vulnerability to embolism in both angiosperms (Crombie et al., 1985; Sperry and Tyree, 1988; Choat et al., 2004) and gymnosperms (Cochard et al., 2009; Hölttä et al., 2012). These experiments have documented that a decrease in σ_xylem_ is proportional to Ψ_stem_ causing a given amount of embolism in accordance with Eq. (1) (**Figure 1A**). In addition, a decrease in σ_xylem_ is expected to lead to a lowered stem water content, as embolism formation empties the water conduits and decreases the capillary forces retaining water in extracellular spaces (Tyree and Yang, 1990) (**Figure 1B**). The surfactants responsible for changes in σ_xylem_ are not well known, although lipid surfactants have recently been found to be naturally present in xylem sap (Schenk et al., 2015; Schenk et al., 2017).

European spruce bark beetle (*Ips typographus*) acts as a vector of ophiostomatoid fungi, of which *Endoconidiophora polonica* is the most virulent pathogen, capable of causing extensive necrotic lesions in infected tissues followed by wilting symptoms of the canopy and eventually tree death (Horntvedt et al., 1983; Krokene and Solheim, 1998; Repe et al., 2015; Linnakoski et al., 2017a; Linnakoski et al., 2017b). Bark beetles bore through bark, enabling the insect-vectored fungi to spread to the cambial zone, where the hyphae rapidly grow in the phloem and through the cell walls to adjacent living cells (Franceschi et al., 1998). The fungal hyphae grow radially in the ray parenchyma cells towards the xylem, where the hyphae spread among the tracheids via the bordered pits (Franceschi et al., 1998). Virulence of *E. polonica* is related to its capability to invade deeper into the xylem than other pathogenic ophiostomatoid fungi (Horntvedt et al., 1983; Krokene and Solheim, 1998). The role of reduced σ_xylem_ in xylem water transport during pathogenic infection, such as *E. polonica*, has previously been hypothesized but remains to be investigated (Tyree and Sperry, 1989; Christiansen and Fjone, 1993; Kuroda, 2005).

The aim here was to investigate the hypothesis of decreasing σ_xylem_ in Norway spruce (*P. abies*) saplings during *E. polonica* infection, focusing on the tree hydraulics perspective at the later stage of infection, i.e. after visible symptoms occur in the xylem of infected trees. The role of σ_xylem_ in tree water transport is not well known, especially during biotic interactions. To the authors’ knowledge, previous studies have not reported actual measurements of σ_xylem_ in infected trees. Our study may elucidate the physiological connection between *E. polonica* and its host tree, providing insight into the possible mechanisms involved in the decrease in tree vitality. We hypothesized that infection will result in (i) a decrease in σ_xylem_ and (ii) embolism formation with a decrease in xylem hydraulic conductivity. Thus, σ_xylem_ and stem hydraulic conductivity (K_stem_) were measured along with canopy conductance (g_canopy_), xylem water potential (Ψ_stem_) and stem relative water content (RWC_stem_). Additionally, analyses of chemical compounds in the xylem sap were conducted to determine whether the concentration of specific compounds would correlate with σ_xylem_.

## Materials and Methods

### Plant Material and Experiment Design

The study was conducted in a greenhouse at the Viikki campus of the University of Helsinki, Finland (60° 13' N 25° 1' E) from June 10 to September 15, 2016. The environmental conditions in the greenhouse were set to day and night temperatures (24±2°C and 20±3°C) and relative humidity (57±10% and 67±10%). The sapling material (approximately 1.2 m in height; diameter above grafting 1.5±0.2 cm) was moved from the Haapastensyrjä nursery, southern Finland (60° 4’ N, 24° 3’ E) to Viikki campus in mid-May 2016. Two grafted clones (64 and 1510) of Norway spruce with 22 saplings in each clone were chosen as plant material for the study. Clones were used for reducing the variation caused by the tree genotypes in the studied tree responses to the *E. polonica* infection. The saplings were immediately replanted in 10-liter pots with a turf and soil mixture, and placed in the greenhouse. The saplings were allocated to three treatment groups: inoculated trees (10 per clone), wounded control trees (10 per clone), and intact control trees (2 per clone) for ruling out the wounding effect. The inoculated trees were inoculated with *E. polonica* (strain F5) (Linnakoski et al., 2017b) growing on 2% Malt Extract Agar (MEA). Inoculation was performed using a sterile 6 mm hole puncher to cut a bark disc, and an inoculum of the same size was placed onto the exposed xylem surface. The inoculation site was then covered with the bark disc and sealed with parafilm. The wounded trees were mock-inoculated following the same protocol, using sterile 2% MEA (later referred as wounded control), and the control trees were left intact. Each sapling was inoculated at approximately 40, 50, and 70 cm above the tree stem base, with the lowest site always above the grafting and all inoculations on the same side of the stem. The experiment with clone 64 began and ended a week before the experiment with clone 1510. Both clones, as well as the different treatments, were randomly distributed in the greenhouse. The saplings were irrigated weekly with approximately the same amount of water lost by transpiration during the week (~ 1 L).

### Physiological Measurements

Physiological and hydraulic measurements are compiled in **Table 1**. Tree water relations were monitored weekly from the second week onwards after inoculation by measuring the whole-tree transpiration rate and Ψ_stem_ above the inoculation point. The transpiration rate was measured gravimetrically by weighing the water loss of the saplings over a few hours, at about the time of the highest transpiration. g_canopy_ was calculated as the sapwood area-specific whole-tree transpiration rate divided by the vapor pressure deficit (VPD). VPD was calculated from temperature and relative humidity, which were measured by a Priva measuring box (Priva, Beijing, China), and the mean VPD over each measurement time was used for the calculation of g_canopy_. Ψ_stem_ was measured simultaneously with the transpiration measurement from detached needle tips with a Scholander-type custom-made pressure chamber. The needles were covered with an aluminum bag for approximately 20 min to stop transpiration before collection of needle tips for Ψ_stem_ measurement (e.g. Turner, 1988).

**Table 1:** Physiological and hydraulic variables studied in *Endoconidiophora polonica*-inoculated and wounded control *Picea abies* saplings.

	Variable	Sampled/measured from	Aim/purpose of measurement
σ_xylem_	Xylem sap surface tension	Xylem sap collected from stem segment	σ_xylem_ is proportional to the xylem water potential that the tree withstands without embolism
K_stem_	Stem hydraulic conductivity	Stem segment (xylem)	Xylem-specific hydraulic conductivity locally in the stem, a decrease indicates embolism
RWC_stem_	Stem relative water content	Stem segment (xylem) adjacent to K_stem_ sample	Considerable decrease in stem relative water content indicates embolism
Ψ_stem_	Stem water potential	Needles (equilibrated to xylem water potential)	Free energy of water, describes the plant water status
Transpiration rate	Whole-tree-level transpiration rate	Living sapling using a balance	Quantifies whole-tree water use
g_canopy_	Canopy conductance^1^	Calculated by transpiration rate/VPD	Indicates stomatal regulation of tree water use
K_stem,saturated_	Saturated stem hydraulic conductivity	K_stem_ stem segment after water saturation	Xylem-specific hydraulic conductivity in the stem when embolized conduits are refilled
PLC	Percentage loss of hydraulic conductivity	Calculated by Eq. 2 using K_stem_ and K_stem,saturated_	Percentage of hydraulic conductivity lost because of embolism
Xylem sap compounds	Concentrations of xylem sap compounds	Xylem sap collected after σ_xylem_ sample	Analyzed for investigating the possible surfactants causing variations in σ_xylem_

^1^i.e. Stomatal conductance at canopy level.

### Sampling 

Saplings were always sampled in pairs (one inoculated and one wounded control at the same time) with two to three repetitions per week altogether for 13 weeks per clone. The first saplings were sampled approximately 3 weeks after the experiment started, starting with clone 64, thereafter sampling one clone every second week. The intact controls were sampled in weeks 6 and 13 following the treatment. The timing of the first samplings was determined in a trial experiment (not shown). This trial study indicated response to inoculation in embolism, detected by ultra-acoustic emission, ca. 2-3 weeks after inoculation.

### Hydraulic Measurements

The uppermost inoculation point was used for the hydraulic conductivity (K_stem_) measurement and the collection of the σ_xylem_ sample. K_stem_ is the hydraulic conductance per given length and cross-sectional area (e.g. within a stem segment). Once a piece of a tree stem (about 10 cm in length) was cut, it was sealed in a plastic zipper bag. Within half an hour from the sampling, bark was peeled from one centimeter of the sample piece end. Silicon tape was wrapped around the peeled part of the stem to ensure water flowed only through the xylem and not through the bark. Stem segment ends were cut under water and attached to tubes with silicon tape, the stem base end to the upper tube with a water column and the stem apex end to the lower tube measuring the water flow rate. Before installing the lower tube, the first water drops coming through the xylem were collected in a plastic tube and used for the σ_xylem_ measurements, apart from the first drop, which was discarded to avoid contamination of the σ_xylem_ sample. K_stem_ was measured by flow of water pushed through sampled stem segments with approximately 0.1 bar pressure (Sperry et al., 1988). The pressure was created by a 1-meter column of water solution with HCl, KCl, and milli-Q water (pH ~ 2.2; osmolality 0.03 mol kg^-1^) through a tube with diameter between 4 and 9 mm according to the diameter of the stem piece. Water flow rate through the stem pieces was measured from the water meniscus with a caliper for 20 min by measuring the water level in the tube in upright position. Thereafter, the volumetric water flow rate through the stem piece was calculated by multiplying with the cross-sectional tube area. In addition to the K_stem_ measurement, the saturated K_stem_ (K_stem,saturated_) was measured after pulling water through the stem pieces at ca. 0.8 bar pressure for 30 min with a vacuum pump (XX5522050, Millipore, MA, USA), and thereafter saturating the stem pieces in milli-Q water for 24 h prior to the measurement. Percentage loss of conductivity was thereafter calculated according to Pammenter and Van der Willigen (1998):

(2) $$\text{PLC}=(1-{\text{K}}_{\text{stem}}/{\text{K}}_{\text{stem,saturated}})*100.$$

The σ_xylem_ measurements were carried out with a capillary rise method using the setup of Vanhanen et al. (2008). Glass capillary tubes were cleaned with sulphuric acid and distilled water before each measurement. The contact on angle of the capillary tubes was expected to be close to zero as it is known to be for clean glass (Cras et al., 1999). Dry capillary tubes were then placed in a holder of the collected samples in plastic tubes at room temperature (20° C) for 1 h. The capillary rise was then measured with a hand-held caliper. The inner radius of the tubes was calibrated using distilled water, which has a well-known surface tension at a specified temperature. σ_xylem_ of the xylem was calculated according to Bikerman (1947) and Vanhanen et al. (2008):

(3) σ=1/2ρgr(h+r/3),

where σ is surface tension (N m^-1^), ρ is water density (kg m^-3^), g is the gravitational acceleration (9.81 m s^-2^), r is the radius of capillary tube (m), and h is the height of capillary rise inside the tube (m). In reality, the density of xylem sap is slightly higher than the density of water; however, xylem sap is such a dilute solution (Borghetti et al., 1991) that we disregarded the effect of dissolved solutes on xylem sap density. Each σ_xylem_ measurement is a mean of two measurements from the same sample.

RWC_stem_ was measured from a separate stem segment adjacent to the highest inoculation point. The fresh weight of a stem segment was weighed after removing the bark. The stem segment was then saturated in milli-Q water for 48 h, and the turgid weight was measured after extra water on the surface was gently wiped away with paper. Thereafter, dry weight was measured after samples were oven dried at 80°C for 72 h. The RWC_stem_ was as follows:

(4) $${\text{RWC}}_{\text{stem}}=(\text{FW}-\text{DW})/(\text{TW}-\text{DW}),$$

where FW is fresh weight, DW is dry weight, and TW is turgid weight. Inoculated and wounded control trees were always measured simultaneously in pairs when K_stem_, σ_xylem_ and RWC_stem_ were measured.

Saturated stem water content was calculated as follows:

(5) Saturated stem water content=(TW-DW)/DW.

### Symptoms of Infection

The spread and damage caused by *E. polonica* were assessed by using calipers to measure lengths and widths of necrotic lesions on the bark and xylem surface after removing the bark. These symptoms were investigated to determine whether the inoculation treatment was successful. The total lesion length (above and below the inoculation) and width in the bark were measured from the inner surface of the bark. A colored area in the xylem cross-section was considered a lesion, and the conical lesion area to total cross-sectional xylem area ratio was calculated based on the tangential width of the lesion, measured at the widest point, and the radial depth of the lesion. The necrotic lesions are commonly used indicators of pathogen colonization (Krokene and Solheim, 1998), although accurate estimates of pathogen spread are challenging with this and other similar commonly used methods (Linnakoski et al., 2017b).

### Chemical Analyses of Xylem Sap

Xylem sap for the chemical analyses was collected immediately after the surface tension sample was collected following the same protocol. The sap samples were stored in separate plastic tubes at –80°C. Samples from the same treatment and clones, collected during a week (two to three samples), were combined and analyzed as one sample. The sap was dried in a freeze-dryer and silylated with 0.5 mL of 20% TMSI-pyridine mixture [TMSI =1-(trimethylsilyl) imidazole]. The dry matter of the sap was analyzed for its chemical composition by gas chromatography-mass spectrometry (GC-MS) analysis by using a HP 6890 GC system equipped with a mass selective detector 5973 and HP-5 capillary column (30 m × 0.25 mm i.d.; 0.25 μm film thickness). Helium was used as carrier gas at a flow rate of 1.5 mL/min. Heptadecanoic acid (C:17), 1-chlorodecane, and m-erythrit were used as internal standards for resin acids, terpenes, and monosaccharides, respectively. The chromatographic conditions for resin acids were as follows: initial temperature 180°C, temperature rate 5°C/min, final temperature 300°C for 5 min, injector temperature 280°C and split ratio 1:20, MS-interface temperature 300°C, and ion source temperature 230°C. Mass spectra were obtained by electron impact (EI mode) ionization energy 70 eV. Monoterpenes and sesquiterpenes were analyzed directly from the liquid sap solution with GC-MS, using the following conditions: initial temperature 30°C, rate 10°C/min, hold time 5 min; rate 40°C/min, hold time 2 min; final temperature 230°C. For monosaccharides, the chromatographic conditions were as follows: initial temperature 110°C; rate of temperature increase 10°C/min; final temperature 320°C maintained for 14 min; injector temperature 260°C, and split ratio 1:20. The MS-interface temperature was 300°C and that for ion source was 230°C. All results were calculated by using an internal standard. Only samples from inoculated and wounded control trees were analyzed, as the sample amount was insufficient in the intact control trees with two intact control individuals per clone.

### Statistical Analyses

Student’s t-test and linear regression were used for statistical testing. Results were considered significant when P-value was less than or equal to 0.05. Statistical testing was carried out using R (v. 3.2.1, 2015, R Foundation for Statistical Computing, Vienna, Austria).

## Results

### Symptoms of Infection

The inoculated trees had larger lesions than the wounded control trees (P < 0.001), accounting for 19% (clone 64) and 23% (clone 1510) of the xylem cross-sectional area at the inoculation site, with width approximately 1.6 cm in the bark of both clones, and total length 7.1 and 9.0 cm in the inner surface of the bark of clones 64 and 1510, respectively (see **Figure S1** in **Supplementary materials**). In the wounded control trees, the lesions were only slightly larger than the inoculation wound (6 mm), covering 6% and 5% of the xylem cross-sectional area at the inoculation point, and measuring 1.0 and 0.9 cm in width and 1.4 and 1.3 cm in length in the bark of clones 64 and 1510, respectively. Additionally, declines in tree vigor and habitus were visible in inoculated trees, e.g. desiccation of shoots above the inoculation was visible in some of the inoculated trees.

### Xylem Sap Surface Tension and Tree Water Relations

**Figure 2:**
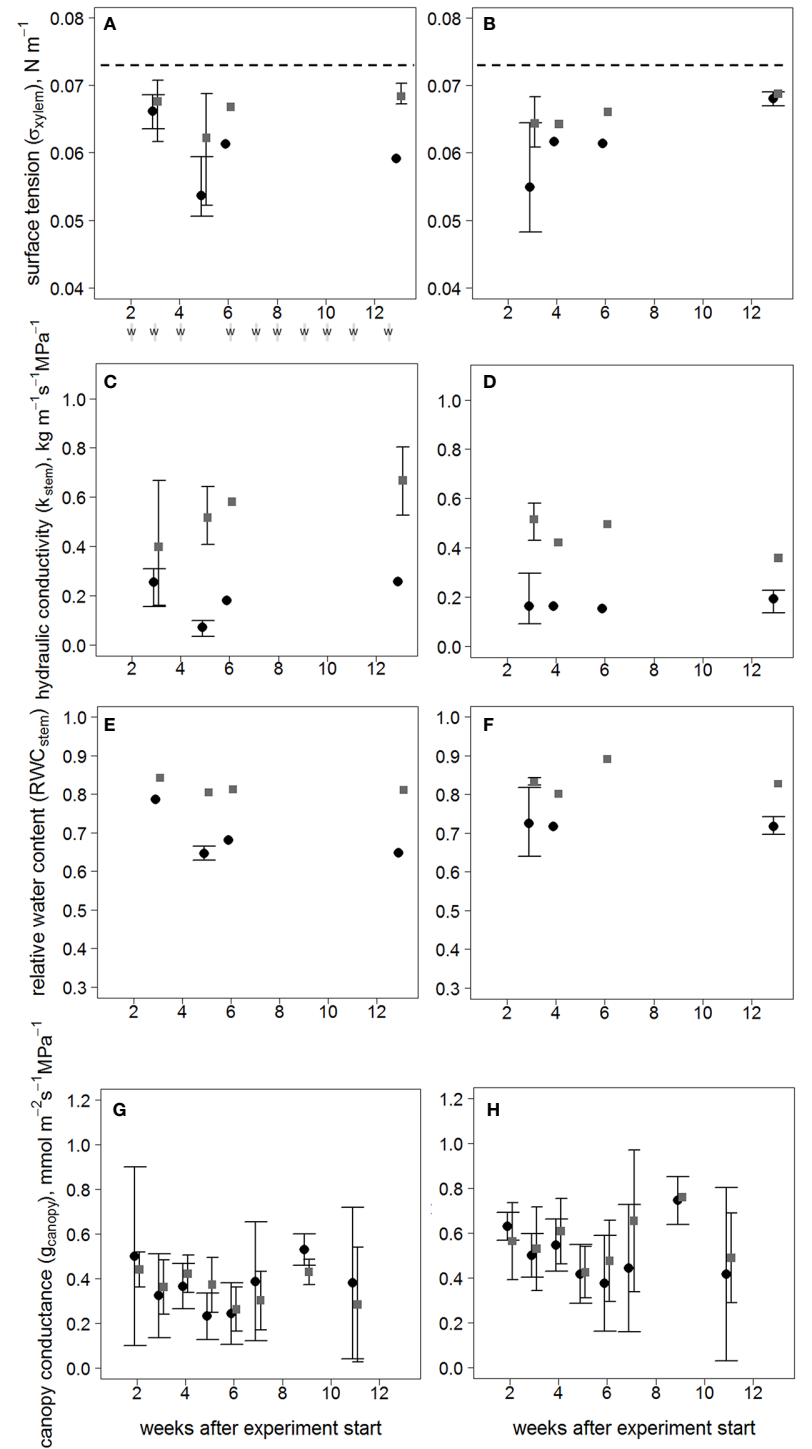
Responses over time following treatment, after visible symptoms of infection have occurred in xylem for σ_xylem_ of clones 64 **(A)** and 1510 **(B)**; K_stem_ clones 64 **(C)** and 1510 **(D)**; RWC_stem_ clones 64 **(E)** and 1510 **(F)**; and g_canopy_ calculated with the sapwood area-specific transpiration rate of clones 64 **(G)** and 1510 **(H)** in *Endoconidiophora polonica*–inoculated and wounded control *Picea abies* saplings. Inoculated trees are shown in black circles and wounded controls in gray squares. Error bars (with wide ticks in inoculated trees and narrow ticks in wounded controls) show the range between the minimum and maximum values (three observations), except in **(G, H)**, where error bars represent standard deviations (n=3–5/treatment/clone), and no error bars are present when only 1-2 trees were measured. Both inoculated trees and wounded controls were sampled simultaneously, and the points are separated in the figure only for clarity sake. Times of irrigation (w) are shown beneath **(A)**. Dashed lines (in **A**, **B**) indicate the surface tension of pure water at 20 °C.

The treatments began to diverge in terms of σ_xylem_ (**Figures 2A, B**) and K_stem_ (**Figures 2C, D**) at the first sampling in clone 1510 three weeks after inoculation, and subsequently in clone 64. The difference in K_stem_ was distinct in both clones towards the end of the experiment. However, the σ_xylem_ of inoculated trees returned to levels similar to the wounded control trees 13 weeks after the inoculation in clone 1510, whereas the σ_xylem_ of inoculated and wounded control trees in clone 64 differed also after 13 weeks. All σ_xylem_ observations differed from the surface tension of pure water (i.e. ~0.073 N m^-1^ at 20°C).

The RWC_stem_ describing the amount of wood desiccation was lower in inoculated saplings than in wounded controls in both clones throughout the experiment (P < 0.01; n = 8/treatment/clone) (**Figures 2E, F**). Neither g_canopy_ (**Figures 2G, H**) nor the Ψ_stem_ values differed between treatments (**Supplementary Figure S2**), indicating that there was no difference in stomatal conductance or water potential between the treatments.

**Figure 3:**
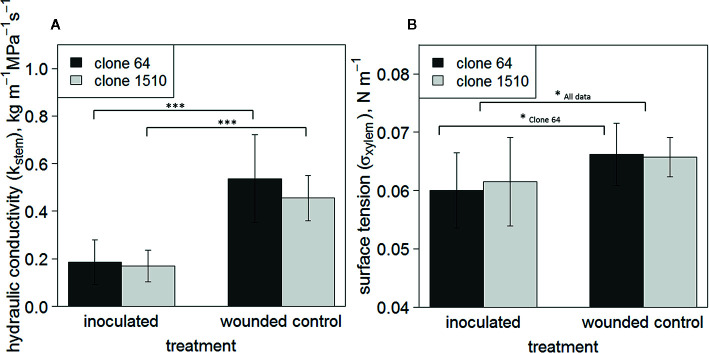
Mean K_stem_ **(A)** and σ_xylem_ **(B)** of clones 64 and 1510. Error bars show standard deviations. Observations include all sampled *Endoconidiophora polonica* inoculated and wounded control *Picea abies* saplings in the experiment (n = 9–11/treatment/clone). Statistically significant differences between treatments are found in K_stem_ (P < 0.001; n = 9–11/treatment/clone), and in σ_xylem_ when all trees are pooled together (P < 0.05; n = 20/treatment) and in clone 64 (P < 0.05; n = 10–11). Statistically significant differences between treatments are indicated by symbols: * P < 0.05; *** P < 0.001. Brackets in **(A)** indicate to statistically significant differences in clones 64 and 1510 separately, whereas in **(B)** the statistically significant difference between treatments is shown for clone 64 and when both clones are pooled together.

Mean K_stem_ was significantly lower in inoculated trees than in wounded controls in both clones (P < 0.001; n = 9–11/treatment/clone) (**Figure 3A**), indicating more embolism formation in the inoculated saplings. Mean σ_xylem_ was also significantly lower in inoculated trees when all observations, i.e. both clones, were examined together (P < 0.05; n = 20/treatment), and in clone 64 (P < 0.05; n = 10–11/treatment) (**Figure 3B**). Statistical significance between the treatment groups was not tested within each individual measurement day due to the limited number of saplings and the destructive nature of the sampling. The mean σ_xylem_ was 0.066 N m^-1^ in the wounded control trees of both clones, and 0.060 and 0.062 N m^-1^ in inoculated clone 64 and 1510 trees, respectively (**Figure 3B**). σ_xylem_ values from clone 64 saplings ranged between 0.051 and 0.069 N m^-1^ (inoculated trees) and between 0.052 and 0.071 N m^-1^ (wounded control). However, only one observation from the wounded control trees was below 0.06 N m^-1^, whereas most of the observations in the inoculated trees were below 0.06 N m^-1^. In clone 1510 saplings, the σ_xylem_ values ranged between 0.048 and 0.069 N m^-1^ (inoculated trees) and between 0.061 and 0.072 N m^-1^ (wounded control). In intact control trees, the σ_xylem_ ranged between 0.065 and 0.066 N m^-1^ (clone 64) and between 0.062 and 0.068 N m^-1^ (clone 1510).

When excluding the data points where K_stem,saturated_ was smaller than K_stem_, the inoculated trees demonstrated higher PLC than the wounded controls (14% vs. 6%) (see **Supplementary Figure S3**), albeit without statistical significance (P = 0.08; n = 9–12/treatment). However, K_stem,saturated_ (P < 0.001; n=13–15/treatment) and saturated stem water content (P < 0.001; n=17–18/treatment) were lower in the inoculated trees (see **Supplementary Figure S4**), indicating that not all conduits could be refilled with water in the inoculated samples, and therefore, the PLC calculations for the inoculated samples represent underestimates of the actual PLC, and the results based on PLC calculations should be treated with caution.

**Figure 4:**
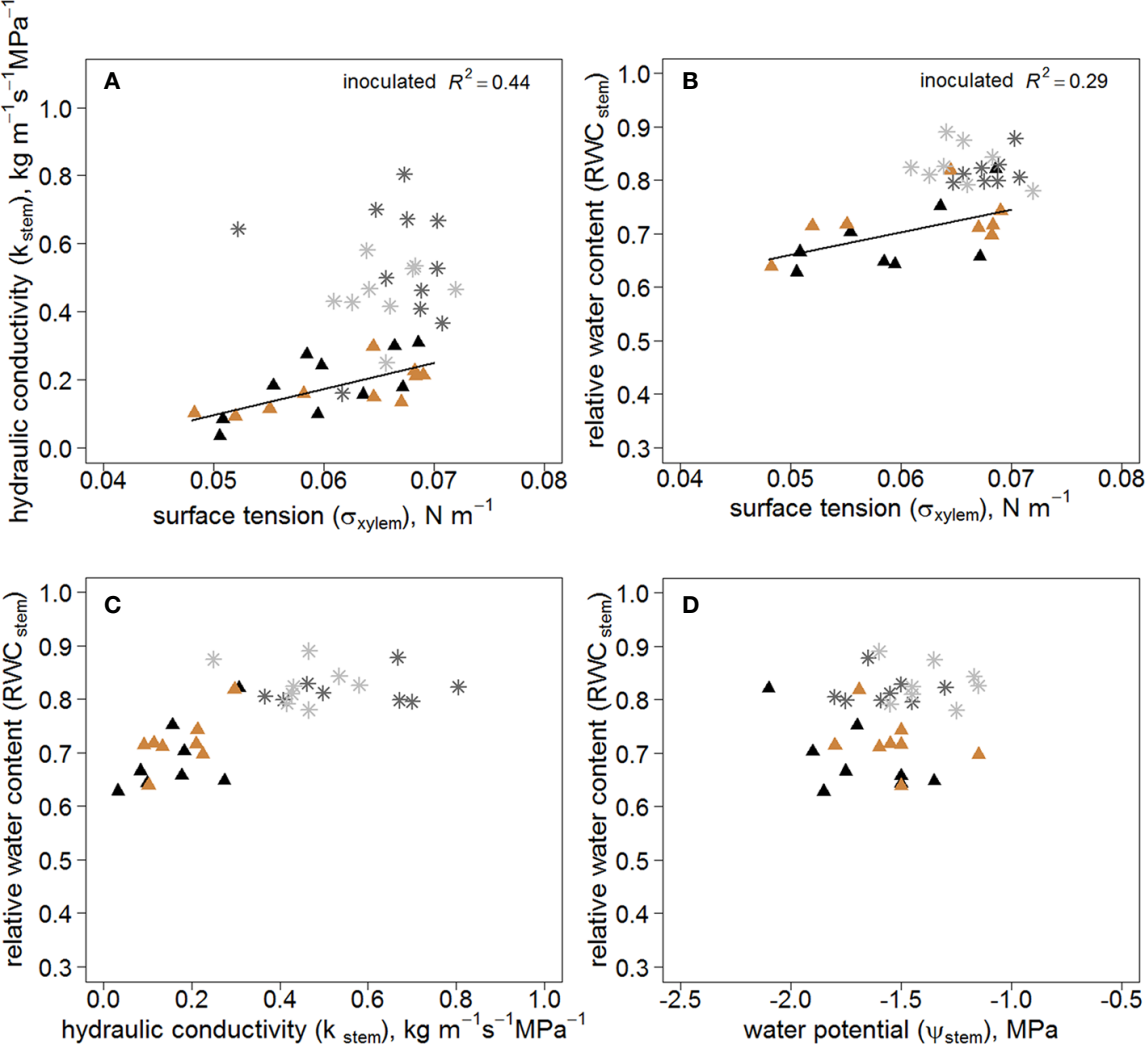
K_stem_ **(A)** (n=20/treatment) and RWC_stem_ **(B)** (n=16/treatment) as a function of σ_xylem_; RWC_stem_ in relation to K_stem_ **(C)** (n=16/treatment) and Ψ_stem_ **(D)** (n=16/treatment) in all measured *Endoconidiophora polonica* inoculated and wounded control *Picea abies* saplings. Inoculated trees are shown in black (clone 64) and light brown (clone 1510) triangles, and the wounded controls in dark grey (clone 64) and light grey (clone 1510) star symbols in the figure. The linear regression equations of σ_xylem_ and K_stem_ **(A)** y=-0.288+7.665x, (P < 0.01); and σ_xylem_ and RWC_stem_ **(B)** y=0.45 + 4.24x, (P < 0.05).

K_stem_ correlated positively with σ_xylem_ in the inoculated trees, whereas there was no correlation in the wounded control trees (**Figure 4A**). Additionally, RWC_stem_ of the inoculated trees correlated with σ_xylem_ (**Figure 4B**). RWC_stem_ remained constant in relation to K_stem_ in wounded controls compared with inoculated trees (**Figure 4C**). RWC_stem_ for a given Ψ_stem_ was lower in inoculated trees (**Figure 4D**).

### Compounds in Xylem Sap

In xylem sap, the total concentration of compounds was larger in inoculated trees than in the wounded controls in only clone 64 (**Table 2**). Increased concentrations of quinic acid and alkylphenol (P < 0.05) were found in xylem sap of inoculated trees in clone 1510. Quinic acid concentration increased in xylem sap, especially in the last measurements after recovery in K_stem_ and σ_xylem_ in inoculated trees in clone 1510 (see **Supplementary Figure S5**). In addition, myo-inositol and sucrose concentrations were significantly higher in inoculated trees (P < 0.05) when both clones were examined together. K_stem_ of inoculated trees correlated negatively with myo-inositol concentrations in the xylem sap (see **Supplementary Figure S5A**). Myo-inositol of xylem sap correlated negatively also with σ_xylem_ (see **Supplementary Figure S5B**). In addition, the inositol isomer correlated negatively with σ_xylem_ (R^2^ = 0.41; P < 0.01) and K_stem_ (R^2^ = 0.58; P < 0.05) (not shown).

**Table 2:** Compounds analyzed from the extracted xylem sap from *Endoconidiophora polonica* inoculated and wounded control *Picea abies* saplings.

Clone treatment	64		1510			
	Inoculation	Wounded control		Inoculation	Wounded control
	Mean ± SD	Mean ± SD		Mean ± SD	Mean ± SD
*Carboxylic acids*					
Malic acid	0.07 ± 0.03	0.09 ± 0.04		0.17 ± 0.04	0.14 ± 0.02
Quinic acid* ^(1510)^	0.05 ± 0.01	0.04 ± 0.02		0.07 ± 0.02	0.04 ± 0.01
*Cyclitols and sugars*					
Pinitol	0.45 ± 0.20	0.22 ± 0.04		0.39 ± 0.06	0.42 ± 0.23
Myo-inositol*	0.03 ± 0.02	0.02 ± 0.01		0.04 ± 0.01	0.03 ± 0.01
Inositol isomer	0.02 ± 0.02	0.01 ± 0.00		0.03 ± 0.01	0.01 ± 0.01
α-glucose	0.24 ± 0.16	0.12 ± 0.03		0.42 ± 0.36	0.41 ± 0.24
β-glucose	0.41 ± 0.27	0.20 ± 0.05		0.72 ± 0.62	0.71 ± 0.39
fructose	0.68 ± 0.56	0.37 ± 0.10		1.08 ± 0.88	1.60 ± 0.67
d-Psicose	0.17 ± 0.14	0.09 ± 0.02		0.32 ± 0.27	0.44 ± 0.23
Sucrose*	1.05 ± 0.60	0.41 ± 0.11		0.79 ± 0.36	0.39 ± 0.33
					
Alkylphenol* ^(1510)^	0.04 ± 0.03	0.04 ± 0.03		0.12 ± 0.06	0.01 ± 0.01
Sum	3.22 ± 0.95	1.61 ± 0.29		4.14 ± 2.46	4.20 ± 1.30

No intact control trees are included since the number of samples was too low. Standard deviations are shown adjacent to mean values. Significant differences in concentrations between the treatments are indicated by *P < 0.05; **P < 0.01; ***P < 0.001. Compound concentration units are mg mL^-1^. Clone number is in parentheses after significance symbols if statistical significance was found in only one of the clones.

## Discussion

Our preliminary study with clonal Norway spruce saplings showed that *E. polonica* inoculation resulted in a significant decrease in σ_xylem_, and hampered water transport in inoculated trees, thus confirming earlier studies (Horntvedt et al., 1983; Christiansen and Fjone, 1993; Kirisits and Offenthaler, 2002; Sallé et al., 2005). The observed significant decrease in σ_xylem_ in the inoculated trees has been previously hypothesized to affect tree water transport during pathogenic infections (Tyree and Sperry, 1989; Christiansen and Fjone, 1993; Kuroda, 2005; Lachenbruch and Zhao, 2019). Our results indicate that inoculated trees experienced hampered water transport locally, which was not observable in whole-tree-level water use. The measured lesions in the bark and xylem indicated that *E. polonica* infection caused visible reactions in the inoculated trees (Lahr and Krokene, 2013) before measurements of both K_stem_ and σ_xylem_ were conducted. Additionally, desiccation of shoots above the inoculation was visible in some of the inoculated trees. *E. polonica* inoculation have been reported to result in canopy desiccation after losing some of the conducting tissue in xylem of *P. abies* in other studies (Horntvedt et al., 1983; Christiansen and Fjone, 1993; Kirisits and Offenthaler, 2002), and in other tree species (Kuroda, 2005). The inoculation treatment in the present study was considered a high dosage for the saplings (Krokene et al., 1999).

*E. polonica* infection resulted in changes in tree K_stem_, σ_xylem_ and RWC_stem_ locally at the site of inoculation in the stem, while there was no clear difference in g_canopy_ and Ψ_stem_. The decrease in local K_stem_ without a noticeable decrease in g_canopy_ may be explained by the tree hydraulic architecture. Most of the resistance to water transport is in the distal parts (i.e. needles and fine roots) and in the soil-to-root interface (Tyree and Zimmermann, 2002). Therefore, a reduction in local K_stem_ would have to be drastic in order to cause a noticeable effect at the whole-plant level. A noticeable effect on water transport in *E. polonica*-infected mature *P. abies* trees has been observed only after a large decrease in K_stem_ (Horntvedt et al., 1983; Kirisits and Offenthaler, 2002). In the present study, the stomata seemed to respond to changes in Ψ_stem_ rather than to the decrease in K_stem_ (see also Hölttä et al., 2012) because there was no difference in g_canopy_ or water potential between the treatments.

We found a decrease in RWC_stem_ approximately 10 cm above the inoculation points. The decrease in K_stem_ and RWC_stem_ both indicate substantial embolism formation in the xylem (Tyree et al., 1994; Domec et al., 2004; Rosner et al., 2019). The higher PLC in inoculated trees also indicates a higher amount of embolism, which also relates closely to a decrease in RWC_stem_ (Rosner et al., 2019). However, the PLC results should be treated with caution since not all embolized conduits are expected to refill in conifers due to e.g. tyloses formation or aspirated pits (Flynn, 2007; Hietz et al., 2008). The saturated stem water content was also consistently smaller in the inoculated trees in our study, indicating that there are challenges in the refilling of xylem conduits and that the PLC was underestimated in the inoculated samples. Thus, if the loss of hydraulic conductivity was caused only by mechanical blockage because of the fungus or host tree defense responses, we would not expect the saturation after the initial hydraulic conductivity measurement to restore the hydraulic conductivity. RWC_stem_ has been reported to decrease above and below the *E. polonica* inoculations also in the sapwood of mature *P. abies* trees (Horntvedt et al., 1983). Kuroda (2005) found that the sapwood of *Picea jezoensis* desiccated more than 20 cm above the inoculation point preceding the hyphae of *E. polonica*. However, the spread of fungus hyphae was not examined in the present study.

The K_stem_ and RWC_stem_ correlated with σ_xylem_ in inoculated trees, but not in wounded control trees, and the RWC_stem_ and K_stem_ were at distinct levels between the treatments, although the Ψ_stem_ values were similar. This is consistent with the theoretical prediction that a decrease in σ_xylem_ will decrease RWC_stem_ and K_stem_ at a given Ψ_stem_ (as depicted in **Figure 1**), i.e. that embolism and desiccation of conduits will start already in milder water stress due to decreased σ_xylem_. Lowering the σ_xylem_ from pure water (0.073 N m^-1^) to 0.055 N m^-1^ would lower the P_50_, the Ψ_stem_ where 50% of the conductivity in water transport is lost because of embolism, from -3.5 (Bartlett et al., 2016, González-Muñoz et al., 2018) to -2.6 MPa in *P. abies* (as depicted in **Figure 1** and Cochard et al., 2009). *E. polonica* has previously been hypothesized to affect the σ_xylem_ and disable its function by causing embolism formation (Christiansen and Fjone, 1993; Kuroda, 2005). Recently, also other studies have reported that σ_xylem_ decreased below that of pure water. Losso et al. (2017) described seasonal fluctuations in σ_xylem_ ranging between 0.054 and 0.067 N m^-1^ in Norway spruce. Moreover, Christensen-Dalsgaard et al. (2011) and Schenk et al. (2017) measured a σ_xylem_ lower than that of pure water in angiosperm species. The majority of our σ_xylem_ results, in both inoculated and control trees, are similar to those of Losso et al. (2017), but in our study the range of values was slightly higher. Hence, also in our study the σ_xylem_ sap differs clearly from that of pure water, which increases the vulnerability of the xylem to embolism formation.

We were unable to identify any specific surfactant compounds that caused the lowered σ_xylem_. We expected to find negative correlations between σ_xylem_ and specific identified surfactant compounds in xylem sap, but found only a significant negative correlation with myo-inositol. Myo-inositol has been noted to increase—not decrease—the surface tension in a solution in earlier studies (Kaushik and Bhat, 1998), and therefore, another mechanism must be responsible for the negative correlations of K_stem_ and σ_xylem_ with myo-inositol concentrations. Myo-inositol is known to be connected to several metabolic processes such as growth, signaling, stress responses, and osmoregulation (Loewus and Murthy, 2000; Meijer and Munnik, 2003; Valluru and Van den Ende, 2011). Thus, rising abiotic or biotic stress may increase the myo-inositol concentrations in xylem sap. Increased quinic acid concentrations, such as those found in inoculated clone 1510 saplings, have previously been observed in grapevines during a bacterial pathogen infection (Wallis and Chen, 2012). Increased sucrose concentration in xylem sap is probably not lowering the σ_xylem_ in our study, as sucrose, as well as glucose, acts contrary to surfactants (Matubayasi and Nishiyama, 2006). Malic acid is known to be a moderate surfactant (Hyvärinen et al., 2006), but its concentrations were not different between treatments, nor was there a clear relation with σ_xylem_. The monoterpene pinene, another candidate compound and involved in local tree defense responses (Baier et al., 2002), decreases surface tension, but the effect is low because of its limited solubility (DeWitt and Makens, 1932). However, pinene or other compounds of resin were not detected in the xylem sap (e.g. Nagy et al., 2000).

It is also possible that the surface tension reduction is not caused by a single compound, but rather by the properties of the solution mixture. The surface tension of a solution may decrease with the increasing solute concentration or with changes in solute composition in a given mixture of compounds, as reported for fatty acids and model humic-like substances in mixtures with inorganic salts (Prisle et al., 2010; Kristensen et al., 2014). Surface tension of a solution is always a function of the solution composition, and thus, the contribution of single compounds is challenging to assess (e.g. Aumann et al., 2010; Kristensen et al., 2014). Therefore, the decrease in σ_xylem_ also in control trees may result from varying amounts of surfactants either in lower concentrations or in a less efficient overall mixture of surfactants in the xylem sap. The different behavior of σ_xylem_ in inoculated and wounded controls may thus result from additional compounds affecting the solution mixture and the σ_xylem_.

The decrease in K_stem_ and RWC_stem_ seemed to be permanent, while σ_xylem_ recovered, at least in clone 1510. This indicates that the embolisms were either not refilled, or other reasons, such as mechanical blockage, prevented the recovery in K_stem_. Loss in K_stem_ may also be initiated from the formation of tylosoids, pit aspiration, embolism, and/or release of epithelial cell content in the apoplast (Yazaki et al., 2017). However, as our sampling began after the visible symptoms in xylem had occurred, we can only speculate about the initial reasons for decreased K_stem_ in the early stage of infection. *E. polonica* has been shown to proceed deeper in the sapwood through the ray parenchyma cells (Franceschi et al., 1998; Kuroda, 2005). The ray parenchyma spanning across the stem, from inner bark to stem pith (Plavcová and Jansen, 2015), provide a route for the spread of fungus hyphae (Franceschi et al., 1998). The disabling of conducting tissue can also be the result of Norway spruce defense reactions, where accumulating compounds block the invaded tissue (Ballard et al., 1982; Christiansen, 1985; Yamada, 1992). Lesions, induced by the fungal infection and host tree responses, hinder the spread of *E. polonica* with structural barriers and more effective resin compounds (Woodward and Pearce, 1988; Solheim, 1991; Krokene et al., 1999; Viiri et al., 2001). The induced structural barriers may, in turn, affect the water-conducting properties of the xylem tissue, apart from the embolism that recovered after water saturation (as shown above with PLC and RWC measurements).

In conclusion, a decrease in xylem sap surface tension, embolism formation, and a decrease in xylem hydraulic conductivity were observed in *E. polonica*-inoculated trees, as we hypothesized. However, as we started our sampling at the later stage of infection, we are unable to pinpoint the chain of events between the host and the pathogen that led to the correlation between xylem sap surface tension and a decrease in xylem hydraulic conductivity. Xylem sap surface tension may have decreased first, which was followed by embolism formation and a decrease in hydraulic conductivity. Alternatively, the host tree may first have induced cellular responses to infection or the pathogen may have first inflicted mechanical damage to e.g. the resin ducts and/or parenchyma cells, which could have released part of their content to the xylem sap and lowered the surface tension. A decrease in xylem sap surface tension would be important for the tree vulnerability to embolism whether it occurs before or after an initial decrease in hydraulic conductivity, as shown by previous studies (Sperry and Tyree, 1988; Cochard et al., 2009; Hölttä et al., 2012). Our results also indicated that part of the desiccated xylem conduits recovered after refilling with water and that not only mechanical blockage caused the decline in stem hydraulic conductivity. Further investigations of pathogen-host interactions are warranted, as the mechanisms and compounds causing the decreased xylem sap surface tension were not identified. Especially, the limited number of sampled trees prevented us from detecting the early stages of disease development. Furthermore, studies with mature trees are needed to investigate our results in natural conditions, as our study was conducted with saplings under greenhouse conditions. For example, the magnitude and efficiency of tree responses may differ between the bark beetle-vectored *E. polonica* infection in natural conditions and sole inoculation of *E. polonica* (Lahr and Krokene, 2013).

## Data Availability Statement 

All datasets generated for this study are included in the article/**Supplementary Material**.

## Author Contributions

TH, RK, JB, YS, and TP conceived the study. TP, KR, AV, and RL were responsible for establishing the study setup. TP measured the tree water relations, measured infection lesions with KR, and prepared the chemical sampling with KR, TJ, and TL. RL and RK helped to interpret the results from the tree pathology perspective. NP and JL were responsible for the interpretation of the surface tension results. TJ and TL planned and conducted the chemical analyses of xylem sap. All authors contributed to the article and approved the submitted version.

## Funding

This work was supported by the Academy of Finland grant nos. 324014, 272041, and 305763, the Finnish Cultural Foundation grant no. 00180821, and the Japan Society for the Promotion of Science (JSPS) KAKENHI (grant no. 26·04395). NLP and JJL gratefully acknowledge funding from the European Research Council (ERC) under the European Union’s Horizon 2020 research and innovation programme, Project SURFACE (grant agreement no. 717022), and the Academy of Finland (grant nos. 308238 and 314175).

## Conflict of Interest

The authors declare that the research was conducted in the absence of any commercial or financial relationships that could be construed as a potential conflict of interest.
