# Supplementary material for: Is Decreased Xylem Sap Surface Tension Associated With Embolism and Loss of Xylem Hydraulic Conductivity in Pathogen-Infected Norway Spruce Saplings?
Source: Front Plant Sci. 2020 Jul 16;11:1090. doi: 10.3389/fpls.2020.01090 (PMC7378778; doi:10.3389/fpls.2020.01090)
Supplement: Supplementary file 1 [file DataSheet_1.docx]

Supplementary Material

# Supplementary Figures

**
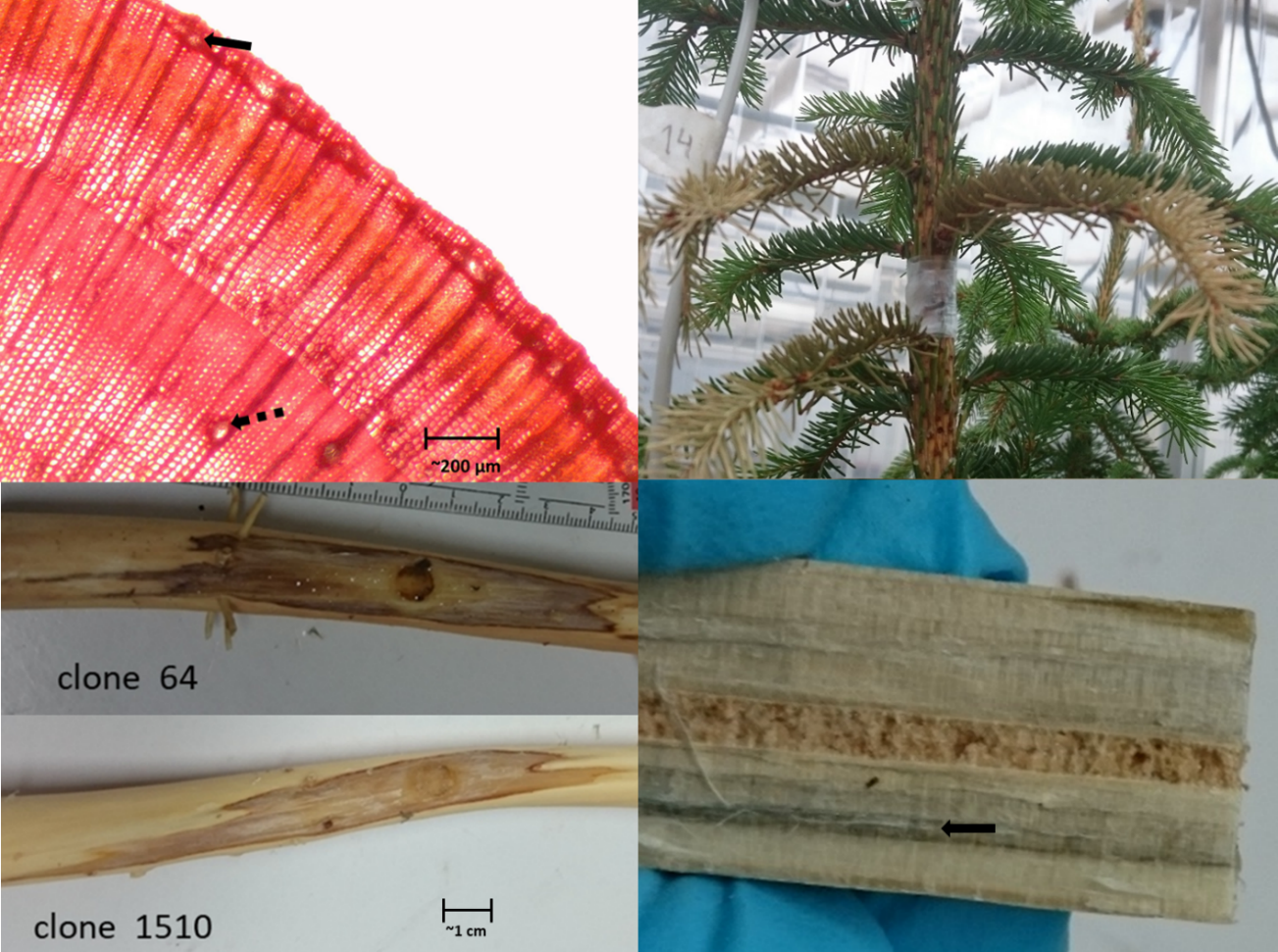
**

**Supplementary Figure S1.** Microscope image of xylem (top left), wilting symptoms in the branches (top right), and lesions in the surface of xylem (bottom left) and arrow indicating the blue-stained xylem (bottom right) in *E. polonica* inoculated *P. abies* saplings. Band of traumatic resin ducts present (solid arrow in top left) and normal resin duct (dashed arrow in top left) in an infected tree. The brown zones, visible in microscope image (top left), may result from released phenolics (Franceschi et al. 2000). These brown areas were not visible in the wounded control trees.


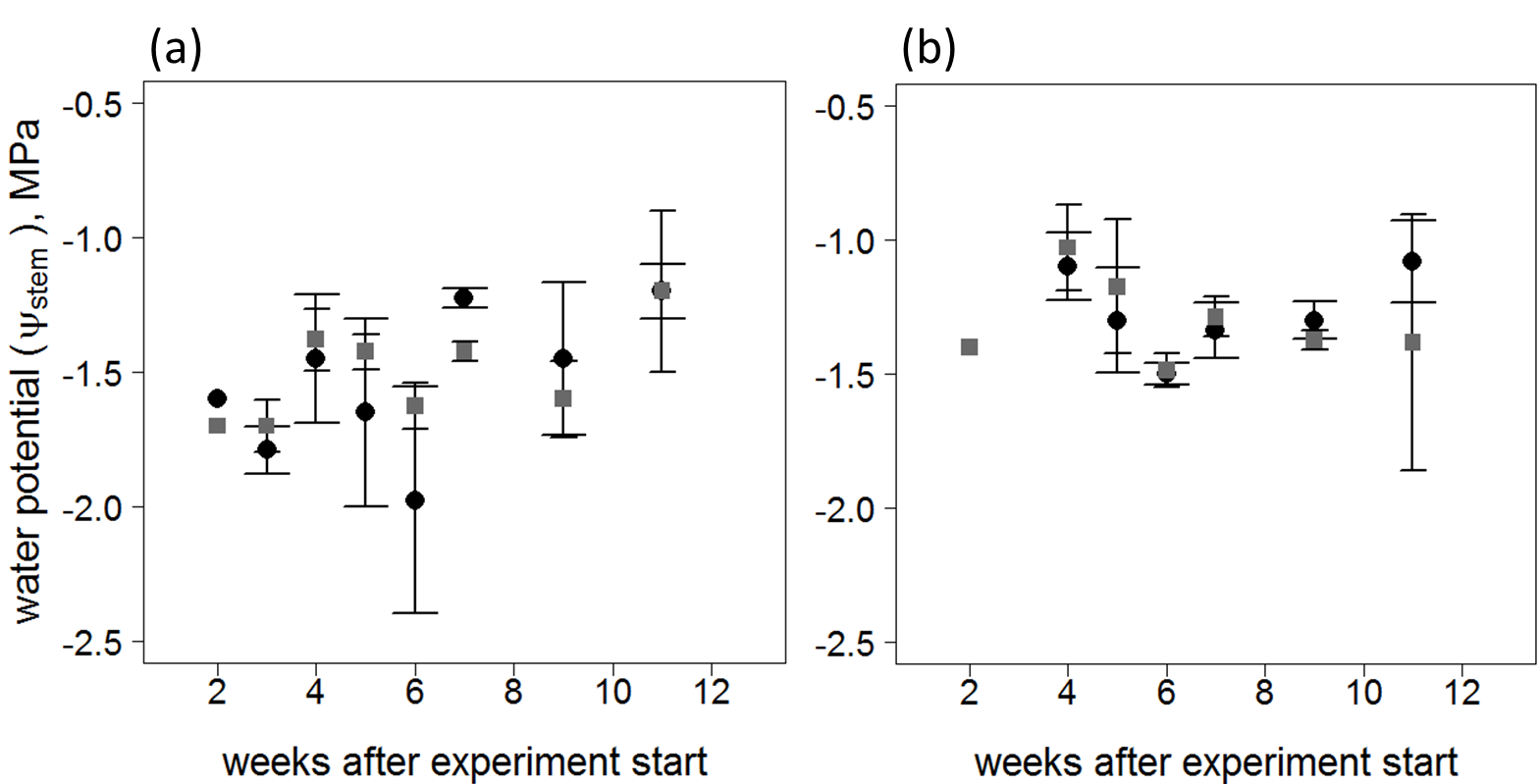


**Supplementary Figure S2.** Responses over time following treatment for Ψ_stem_ of clones 64 (a) and 1510 (b) in *E. polonica* inoculated (black circles) and wounded control (grey squares) *P. abies* saplings. Error bars (with wide ticks in inoculated trees and narrow ticks in wounded controls) show the range between the minimum and maximum values (two to three observations).


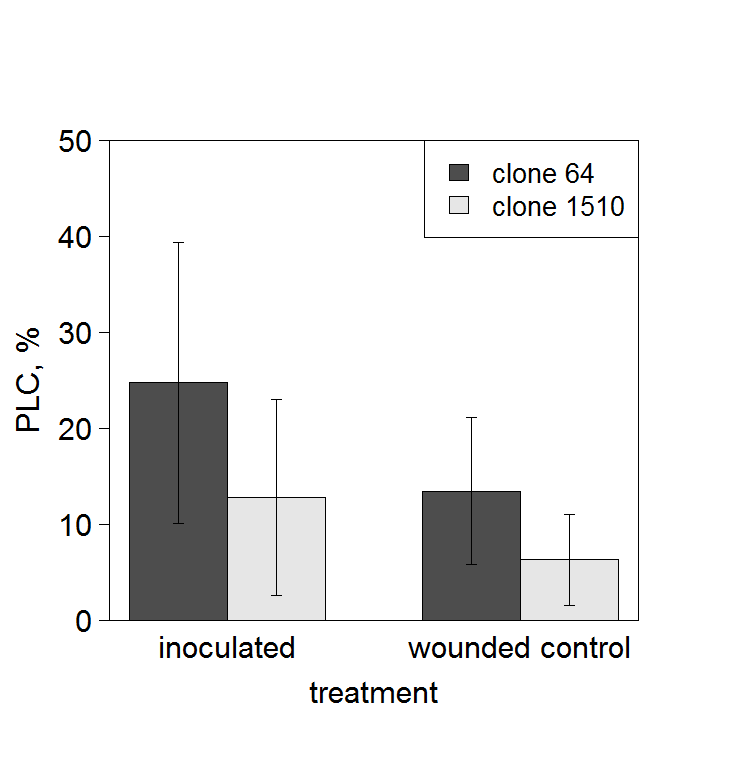


**Supplementary Figure S3.** Percent loss of conductivity (PLC) in clones 64 and 1510 in *E. polonica* inoculated and wounded control *P. abies* saplings (P = 0.08; n = 9-12 / treatment). Error bars are standard deviations.


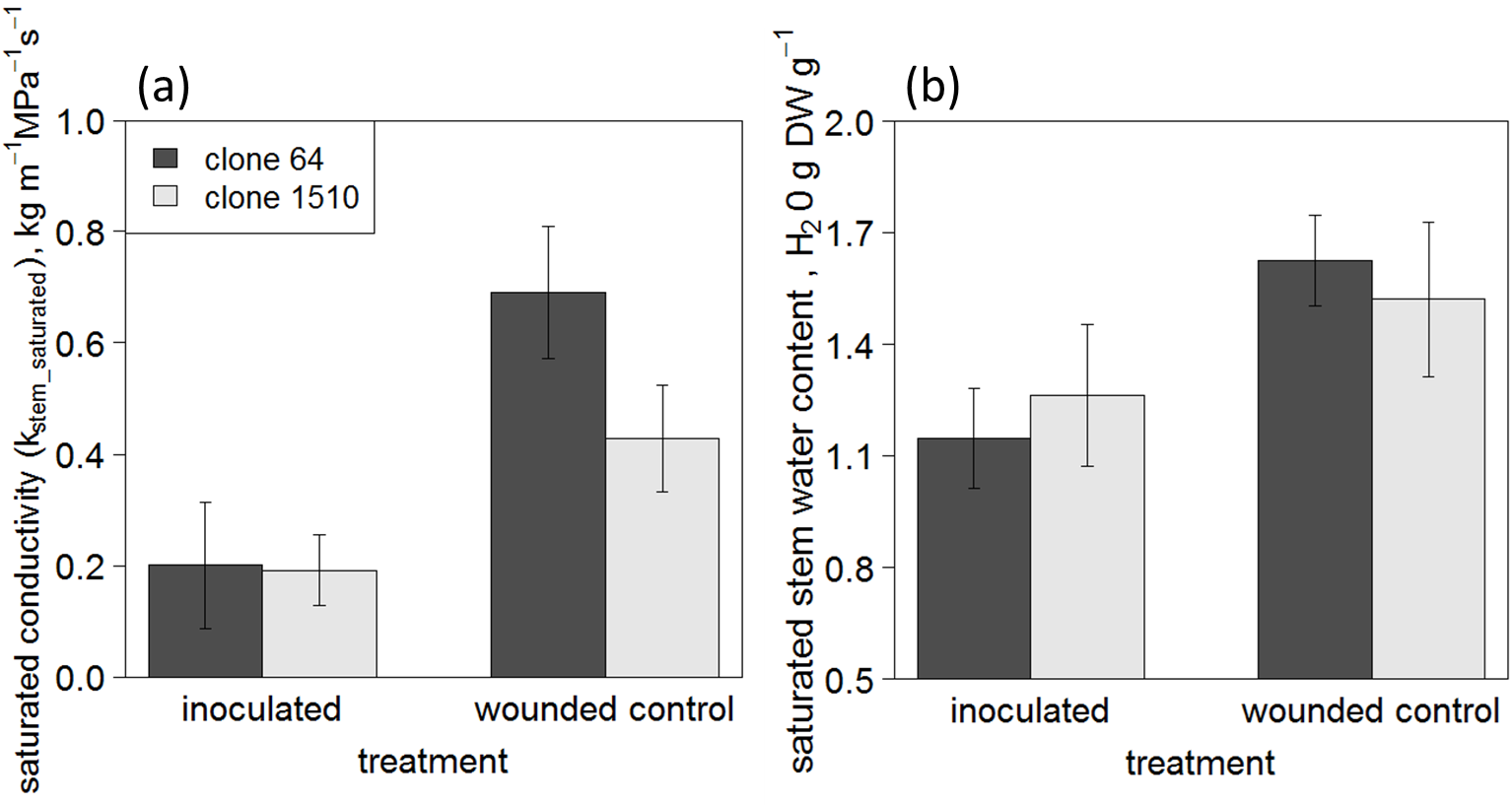


**Supplementary Figure S4.** Saturated stem hydraulic conductivity (K_stem,saturated_) (a) (P < 0.001; n=13-15 / treatment) and saturated stem water content (b) (P < 0.001; n=17-18/ treatment) in clones 64 and 1510 in *E. polonica* inoculated and wounded control *P. abies* saplings. Error bars are standard deviations.

**
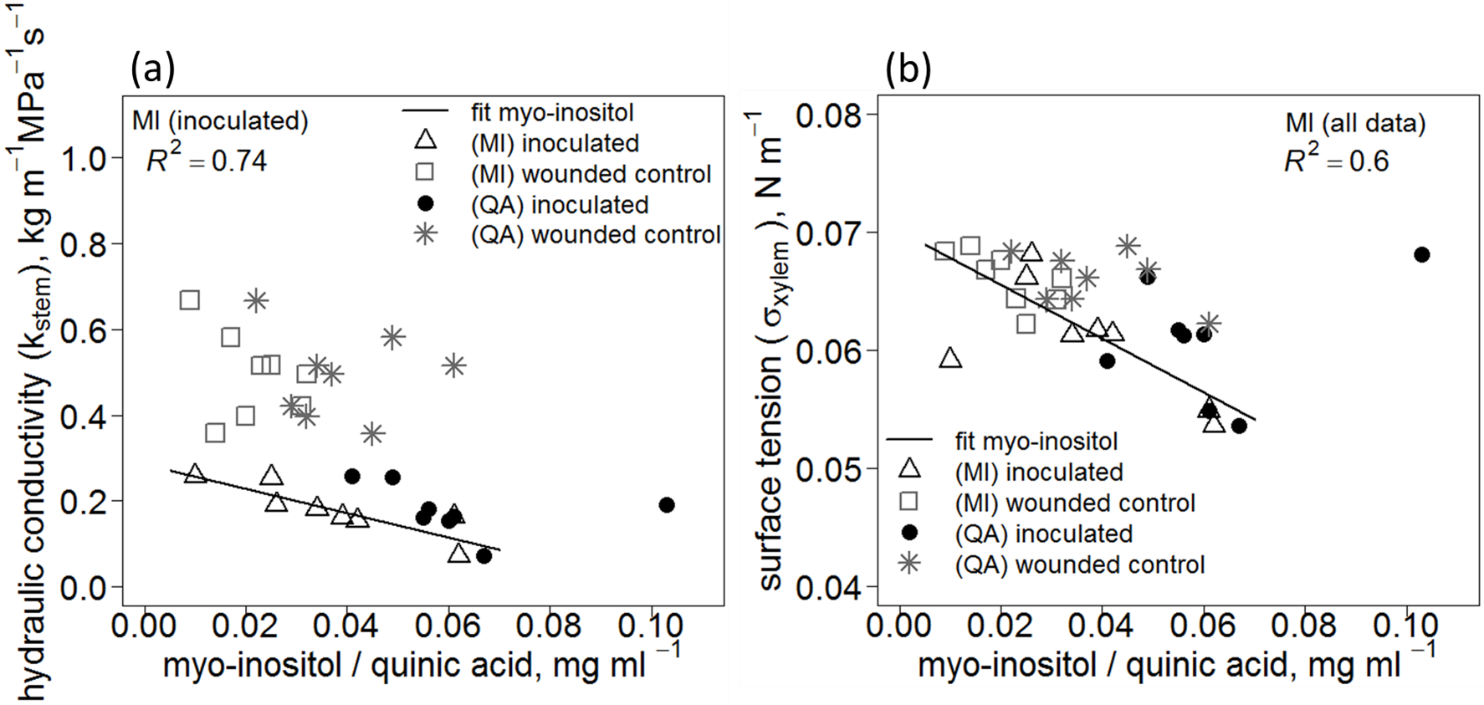
**

**Supplementary Figure S5.** Myo-inositol (MI) and quinic acid (QA) analyzed from the xylem sap in relation to K_stem_ (a) and σ_xylem_ (b) in *E. polonica* inoculated and wounded control *P. abies* saplings of both clones. Statistically tested P-values are for the relations of K_stem_ and myo-inositol (P < 0.01; n=8); and for σ_xylem_ and myo-inositol (P < 0.001; n=16). The highest value in quinic acid concentrations in the inoculated trees is measured from the sap samples that were collected in the last week of measurements, in week 13.
